# Notes on Calomel

**Published:** 1899-02

**Authors:** R. T. Gott

**Affiliations:** Poolesville, Md.


NOTES ON CALOMEL.—To the Medical Council: In malarial fever, quinin will have much better effect if preceded by calomel, gr. x, followed by Epsom salts.

Calomel, gr. x, followed by Epsom salts, will frequently abort an incipient attack of pneumonia. Calomel, Dover's powder and bismuth is the best prescription you can give the baby with fever caused by dentition or indigestion.

When la grippe first visited us it so much resembled malarial fever that I treated it just as I would a case of the latter. It succeeded so well that I have never changed my treatment.

Calomel, gr. x, followed by Epsom salts, is good treatment for dysentery.

To an adult I never give calomel alone. I always rub up opium, gr. ¼, with each dose, and always follow, if necessary, with a brisk purgative—I prefer Epsom salts. In treating a case of malarial fever, I first give the calomel; if it does not move the bowels freely in about eight hours, I give salts; immediately after the salts I begin the quinin, giving gr. v every two hours until the patient is cauchonized, then often enough—about every four hours—to keep up the effect for about twenty-four hours. Then my patient is usually well enough to be discharged.—R. T. GOTT, M.D., Poolesville, Md.

